# Visual training in hemianopia alters neural activity in the absence of behavioural improvement: a pilot study

**DOI:** 10.1111/opo.12584

**Published:** 2018-10-24

**Authors:** Stephanie J Larcombe, Yuliya Kulyomina, Nikoleta Antonova, Sara Ajina, Charlotte J Stagg, Philip L Clatworthy, Holly Bridge

**Affiliations:** ^1^ Oxford Centre for Functional MRI of the Brain (FMRIB) Wellcome Centre for Integrative Neuroimaging University of Oxford Oxford UK; ^2^ Nuffield Department of Clinical Neurosciences (NDCN) University of Oxford Oxford UK; ^3^ Bristol Medical School: Translational Health Sciences University of Bristol Bristol UK; ^4^ Department of Psychiatry Oxford Centre for Human Brain Activity Wellcome Centre for Integrative Neuroimaging University of Oxford Oxford UK

**Keywords:** hemianopia, motion discrimination, perceptual learning

## Abstract

**Background:**

Damage to the primary visual cortex (V1) due to stroke often results in permanent loss of sight affecting one side of the visual field (homonymous hemianopia). Some rehabilitation approaches have shown improvement in visual performance in the blind region, but require a significant time investment.

**Methods:**

Seven patients with cortical damage performed 400 trials of a motion direction discrimination task daily for 5 days. Three patients received anodal transcranial direct current stimulation (tDCS) during training, three received sham stimulation and one had no stimulation. Each patient had an assessment of visual performance and a functional magnetic resonance imaging (fMRI) scan before and after training to measure changes in visual performance and cortical activity.

**Results:**

No patients showed improvement in visual function due to the training protocol, and application of tDCS had no effect on visual performance. However, following training, the neural response in motion area hMT+ to a moving stimulus was altered. When the stimulus was presented to the sighted hemifield, activity decreased in hMT+ of the damaged hemisphere. There was no change in hMT+ response when the stimulus was presented to the impaired hemifield. There was a decrease in activity in the inferior precuneus after training when the stimulus was presented to either the impaired or sighted hemifield. Preliminary analysis of tDCS data suggested that anodal tDCS interacted with the delivered training, modulating the neural response in hMT+ in the healthy side of the brain.

**Conclusion:**

Training can affect the neural responses in hMT+ even in the absence of change in visual performance.

## Introduction

The primary visual cortex (V1) is the first cortical visual area to receive information from the retina via the thalamus. Damage to this area means that the majority of visual input to the brain is lost, leading to full or partial cortical blindness. Although patients with V1 damage show considerable vision loss when tested with standard perimetry, many are still able to detect and respond to information about visual stimuli presented within the blind region, known as ‘blindsight’ or residual vision.^1^, ^2^, ^3^

Since the geniculo‐striate pathway is not functional in patients with V1 damage, there must be an alternative route by which visual information can travel to the brain to facilitate blindsight.^1^, ^4^, ^5^ Recent work has provided evidence that a pathway between the lateral geniculate nucleus (LGN) and motion area hMT+ may underlie these abilities.^6^, ^7^, ^8^, ^9^ Specifically, it has been shown that such a pathway is consistently present in patients who show blindsight, but not those without blindsight.^8^

Visual perceptual learning improves performance in a variety of different tasks. It can occur in those with normal visual performance^10^ and in those with visual disorders such as amblyopia.^11^, ^12^ Several approaches have been taken to try to improve visual performance in cortical blindness, including attempting to strengthen the residual visual pathways that bypass V1. Two main types of rehabilitation training have been used: one involves presenting sinusoidal gratings repeatedly in the blind region of the visual field,^13^ and the other uses moving dot patterns.^14^, ^15^, ^16^ Improvements in visual performance have been reported with both protocols, in the latter case accompanied by an increase in cortical activity in response to stimulation of the blind field.^16^ However, both these approaches require a considerable time investment by the patients; at least 3–6 months of training around five times per week.

We tested whether it was possible to improve visual performance in patients with hemianopia over a considerably shorter period of 5 days, when training was paired with adjunct brain stimulation. The protocol was a motion direction discrimination task that led to significant improvement over this 5‐day period in healthy control participants.^17^, ^18^ This was combined with sham or anodal transcranial direct current stimulation (tDCS) to try to maximise any training effects. Functional magnetic resonance imaging (MRI) was used before and after training to quantify any differences in neural activity that might be induced by visual training or tDCS.

## Materials and methods

### Patients

Seven patients (three female) were included in this study, all of whom had sustained a stroke involving a unilateral lesion to the primary visual cortex which resulted in homonymous hemianopia or quadrantanopia. For inclusion in the study, patients must have sustained the damage a minimum of 6 months before commencing the study to minimise the potential for spontaneous recovery.^19^ Written, informed consent was obtained from all participants and the research adhered to the Declaration of Helsinki. Ethical approval was provided by the South Central Oxford B Research Ethics Committee (15/SC/0483). All participants had normal or corrected‐to‐normal vision, no lateralised visual neglect and no other neurological or eye disease.

### Visual perceptual learning protocol

#### Training

Participants completed training of a motion direction discrimination task *only in the impaired hemifield* each day for five consecutive days, as in previously reported protocols.^17^ Participants were required to determine whether white coherently‐moving dots (luminance 96.8 cd m^−2^) had leftwards or rightwards motion, when displayed amongst randomly‐moving distractor white dots (‘noise’) on a black background (luminance = 0.92 cd m^−2^). The exact location of the stimulus was modified depending on the patient's visual field defect, but was presented entirely within the blind field, within a circular area 8° diameter centred 10–14° to the left or right of fixation. The dot diameter was 0.15°, and the dots moved with a speed of 6°/s for a limited lifetime of 200 ms (12 frames), at a density of 1.5 dots per degree^2^. Dots were randomly reborn at non‐overlapping locations within the stimulus aperture. Coherent motion direction was variable across trials but restricted to within a 90° angle centred around the horizontal meridian.

The task was self‐paced, and participants were given the option of verbal responses or manual responses by way of keyboard arrows. Following each trial, participants received visual feedback as to whether they correctly identified the direction of coherently‐moving dots. A pause screen was implemented every 20 trials to reduce fatigue, and the participants could restart whenever ready.

Task difficulty was modulated according to a two‐up, one‐down adaptive staircase procedure.^20^ Two consecutive correct responses resulted in an increase in the task difficulty by reducing the percentage of coherently‐moving dots by a factor of 0.8. An incorrect response decreased task difficulty by increasing the percentage of coherently‐moving dots by a factor of 1.5. The motion direction discrimination threshold was calculated each time the motion task program was run, by averaging the reversal values (coherence percentage when task difficulty peaks or troughs). The task always began with 80% of the dots moving coherently, and the first 10 reversals were discarded.

Six of the seven patients completed 400 trials of the task each day, while one (Patient 5) only completed 200 trials of the task per day due to fatigue and concentration issues.

**Figure 1:**
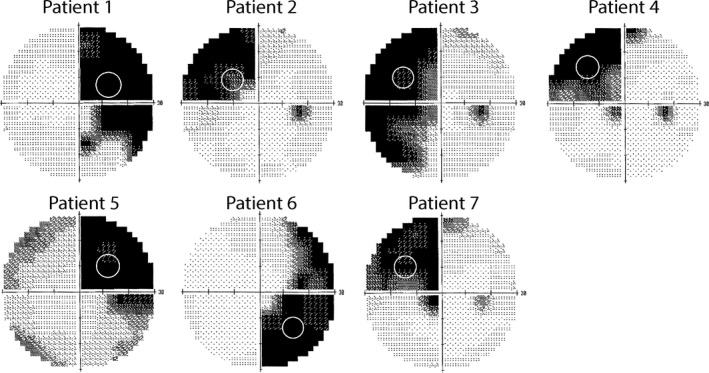
Visual fields for the left eye of each of the patients. The white circle indicates the location at which the stimulus was placed.

The Humphrey visual fields for each patient are shown, along with the location of the training stimulus, in *Figure* 1.

#### Assessments

Participants completed the motion direction discrimination task before and after training as an assessment. The assessment was the same as the training except that no feedback was provided. Additionally, participants were assessed on the motion task in their seeing hemifield, at the equivalent vertical and horizontal offset. Each assessment consisted of 200 trials of the task, and fixation was monitored throughout.

In addition to the motion direction discrimination task, participants were assessed on their motion detection ability before and after training.^7^ In this task participants completed a two‐interval forced choice task, where they were asked to identify the interval in which a white moving dot stimulus appeared. Participants were told to guess if they did not detect a stimulus. Onset of the first interval was indicated by a 500 ms auditory tone at 300 Hz, and the second interval was demarked by a 500 ms 1200 Hz tone. Visual stimuli appeared for 500 ms with jittered onset while the participant fixated on a central white cross. Dot size and density were as described above. Dots were presented at four different speeds: 4°/s, 8°/s, 20°/s or 32°/s in addition to a static condition. The different speeds were presented pseudo‐randomly. Participants were assessed in the blind field, and the equivalent (mirror‐image) location in the seeing field, with 100 trials of each location completed before and after training.

During training, visual fixation was monitored using an EyeLink 1000 eye tracker (http://www.sr-research.com) in five of the seven patients (1, 2, 3, 4, 7) and fixation breaks more than 1° occurred on less than 5% trials. In the assessments, difficulties maintaining a stable signal with the eye tracker meant that only three patients (1, 6, 7) showed stable fixation traces. Patient 4 appeared to make eye movements of around 10° during the assessment periods, and has therefore been highlighted in the behavioural data, as the data from this participant are outliers from the rest of the data. This patient, however, did not make such eye movements during training sessions.

### MRI scanning procedure

Participants were scanned before and after training. Inside the scanner, participants were shown a moving dot stimulus presented in a location as close as possible to the one used in training and its equivalent (mirror‐image) location in the seeing hemifield. The coherence percentage was not adaptive, but rather presented in blocks of 0% (noise), 12.5%, 25%, 50%, 75% and 100% coherence, pseudo‐randomly. Each block lasted for 16 s and the dot motion direction changed every 2 s within each block. Blocks with only the fixation cross present were used as ‘rest’ blocks. The stimulus parameters were otherwise identical to the training parameters.

Participants were asked to respond to a fixation task inside the scanner to ensure the stimulus was delivered to the correct area of the visual field. A white fixation cross flashed red for 200 ms in 25% of trials, and participants were instructed to push a button on a button box each time the flash was detected.

In each scan run, patients were presented with the six block types in each hemifield individually, plus a ‘rest’ block (13 unique block types). Each block type was presented three times per scan run, giving a total of 39 blocks lasting 624 s. Each patient had two scan runs both before and after training.

### MRI data acquisition

All MRI data were acquired using a Siemens MAGNETOM Skyra 3T MRI scanner (http://www.healthcare.siemens.co.uk) with a 32‐ channel head coil at the Clinical Research and Imaging Centre (CRIC), University of Bristol, UK. T1‐weighted structural images were acquired in each scan session at 0.9 mm isotropic resolution (TR = 1800 ms, TE = 2.25 ms, TI = 800 ms, flip angle = 9°). A gradient‐echo echo‐planar imaging (GRE‐EPI) sequence was used to acquire 336 volumes per scan run (36 transverse slices, 3 mm isotropic voxels, TR = 2500 ms, TE = 30 ms; flip angle = 90°); two runs were acquired each scanner visit.

Where possible, fixation was monitored in the scanner with an EyeLink 1000 eye tracker, although consistent recordings were only possible in three of the patients (3, 4, and 7). Note that Patient 4 who made saccades during the behavioural assessment did not make eye movements during the scan sessions and therefore has been included in the analyses. Fixation breaks occurred in <5% of the trials on average and there was no significant difference between fixation breaks pre‐ and post‐training (paired *t*‐test; *t* = 0.5; d.f. = 5; *p* > 0.05).

### Transcranial direct current stimulation (tDCS)

Six patients underwent sham or anodal transcranial direct current stimulation (tDCS), while Patient 3 requested no tDCS. Patients 1, 6 and 7 were randomly allocated by a researcher not involved with the data acquisition to receive the anodal tDCS while Patients 2, 4 and 5 were allocated sham tDCS. The experimenter conducting the training and stimulation was blinded as to whether the participant was receiving sham or anodal stimulation. The stimulation groups were unblinded once data collection was completed, prior to analysis.

Every patient received five 20‐min sessions of tDCS (Neuroconn DC stimulator Plus), delivered over hMT+ in the lesioned hemisphere. One 20‐min session was delivered each day of the training period. For sham stimulation, the current was increased to 1 mA over 10 s and then switched off. For anodal stimulation, the current was increased to 1 mA over a duration of 10 s and remained at 1 mA for 20 min. Direct current was delivered through 5 × 7 cm electrodes inside rectangular saline‐soaked sponges. The cathode was placed at the vertex and the anode was placed 3 cm above the inion along the mastion‐inion line and 6 cm left or right laterally to the midline in the sagittal plane. This setup has been used to successfully stimulate left hMT+ in published literature,^21^ and was also guided by previous brain stimulation studies of hMT+.^22^, ^23^

### MRI data analysis

MRI data analyses were carried out using the Oxford Centre for Functional MRI of the Brain (FMRIB) expert analysis tool (FEAT) v6, which is part of the FMRIB software library, FSL v6 (http://www.fmrib.ox.ac.uk/fsl). Pre‐processing of images included motion correction using MCFLIRT^24^ and spatial smoothing of full‐width half‐height of 5 mm. Magnetic field unwarping (echo spacing = 0.56 ms, EPI TE = 30 ms, unwarp direction = −*y*, signal loss threshold = 10%) and slice timing correction (interleaved) were also applied.

T1‐weighted images were brain‐extracted using FMRIB's brain extraction tool (BET^25^). Functional images were registered to T1‐weighted structural images for each participant, using FMRIB's linear image registration tool.^24^, ^26^ All images were checked to ensure that the lesion had not prevented the brain extraction and registration from working. Lesion size was extracted by manual delineation on the T1‐weighted image.

### Analysis at the whole brain level

Time series statistical analysis was performed using a general linear model (GLM). For whole brain analyses, *z* (Gaussianised T/F) statistical maps of the change in BOLD activity were thresholded using clusters at *z* > 2.3 and a corrected cluster significance threshold of *p* = 0.05. Clusters were projected onto structural space for each participant, and standard space for group analysis.

The first analysis was designed to investigate the overall effect of the training regardless of tDCS status. To enable a group analysis, the brain images of patients with the lesion in the left hemisphere were flipped before registration so that the lesion always appeared in the right hemisphere. Patients 1, 5 and 6 were therefore flipped prior to the first level analyses. A whole brain GLM analysis was performed for each scan run and subject. Each of the 12 block types were entered as explanatory variables (EVs) in the design matrix and linear contrasts between the EVs were computed. A higher‐level fixed effects analysis was then carried out for each subject, to combine data across the two runs per scanner visit. The contrast of coherent stimulus motion compared to noise for each subject for Scan 1 (before training) and Scan 2 (after training) was extracted for the group stage analysis (no other contrasts were analysed further). The lesions were not excluded, but did not affect the group registration or activity.

For group analysis, images were registered to standard space, using the Montreal Neurological Institute (MNI) standard brain with 2 mm isotropic voxels, using FMRIB's nonlinear image registration tool (FNIRT^27^). To determine whether there was an effect of training, a paired t‐test was performed on the first level data from the first and second scan sessions, and compared across participants using a mixed‐effects analysis. Patient 5 showed a considerable amount of motion in both scan sessions (>4 mm) so was excluded from the group analyses, although this did not qualitatively change the results.

In the exploratory analysis designed to investigate potential effects of tDCS on the BOLD signal, the difference in BOLD signal between the pre‐ and post‐training scan sessions was extracted for each patient. A further analysis contrasted the difference between the three patients who received tDCS and those who received sham or no stimulation. Since the power of this analysis is very low given the number of patients in each group, the results of the analysis should be treated as very preliminary, and thus with caution.

### Region of interest (ROI) analysis

A region of interest (ROI) analysis was carried out to calculate the percentage BOLD change in motion area hMT+. This analysis was performed separately for Scan 1 and Scan 2 for every subject, using the mean change in activity when subjects were viewing coherent trials vs motion noise trials. A mask was created of the Juelich‐defined V5 (referred to as hMT+ in this paper) for each hemisphere. The full‐sized Juelich masks indicated where any of the 10 individuals used to generate the atlas showed histological evidence of the region in question.^28^ FEATQuery was used to convert the contrast of parameter estimates (COPE) into the percentage BOLD change, for both scan sessions for each patient.

## Results

### Five days of visual motion training had no effect on performance

**Figure 2:**
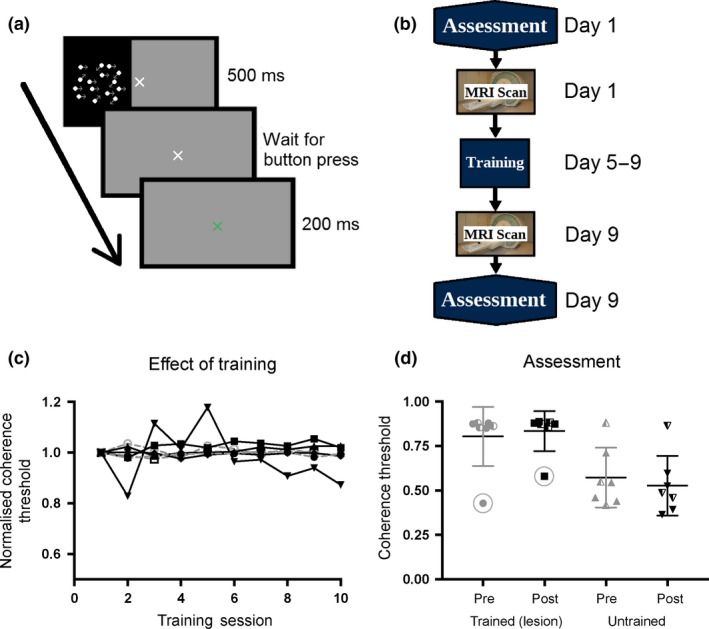
Training protocol and visual stimulation. (a) shows a single trial from the training regime, where the motion stimulus is presented in one hemifield for 500 ms before the subject response. A 200 ms blank screen follows the response before the onset of the next stimulus. (b) shows the protocol followed by each patient, with the initial assessment and MRI scan session on Day 1, training sessions daily on Days 5–9 and the final assessment and MRI scan on Day 9, a minimum of 2 h after the final training session. None of the patients showed any improvement over the 5 days of visual motion training (c). Patients receiving anodal tDCS are shown in grey. The performance was normalised to the coherence threshold of the first training session; if there was an improvement, thresholds would decrease and therefore normalised performance would decrease on the graph, but all patients remain constant around 1.0. (d) shows the coherence threshold measured at the pre‐ and post‐training assessments in both the lesion and intact hemifield. Error bars show standard deviations. Data from patients who received anodal tDCS are shown with the half‐filled symbols. The circled data points are from Patient 4, highlighted due to this participant's frequent breaks of eye fixation during assessment.

The seven patients all undertook daily training on the visual task. Six participants performed two 15‐min sessions per day (400 trials per day) and Patient 5 performed a single session each day (200 trials per day). None of the patients showed any improvement in performance over the 5 days of training, irrespective of whether tDCS was real or sham and therefore all results were combined. *Figure* 2 *c* shows the training performance normalised to the first session. Unlike healthy control participants, who improve over time using exactly the same paradigm,^17^, ^18^ the graph indicates that no patient showed any change in performance with training. Patients were assessed on their performance in both the intact and lesioned hemifields before and after the training sessions. *Figure* 2 *d* shows the coherence thresholds for each of these conditions. Not surprisingly all patients performed better in the sighted hemifield than the blind hemifield (pre‐training: *t*_6_ = 3.0; *p* = 0.02; post‐training: *t*_6_ = 3.9; *p* = 0.008). There was no difference between the pre‐ and post‐training performance in the impaired hemifield (t_6_ = 1.4; N.S.). Similarly, there was no difference in the intact hemifield, *which did not receive any training* (*t*_6_ = 0.73; N.S.). Data from patients who received the anodal tDCS are shown with the half‐filled symbols, and are indistinguishable from the patients who received either sham or no stimulation. The performance of Patient 4, identified with the circles, is significantly better in the blind field than all other patients. However, this is likely explained by eye movements the patient made during the visual assessment, identified from eye tracker traces.

### Only a few patients showed activation of hMT+ in the lesioned hemisphere

**Figure 3:**
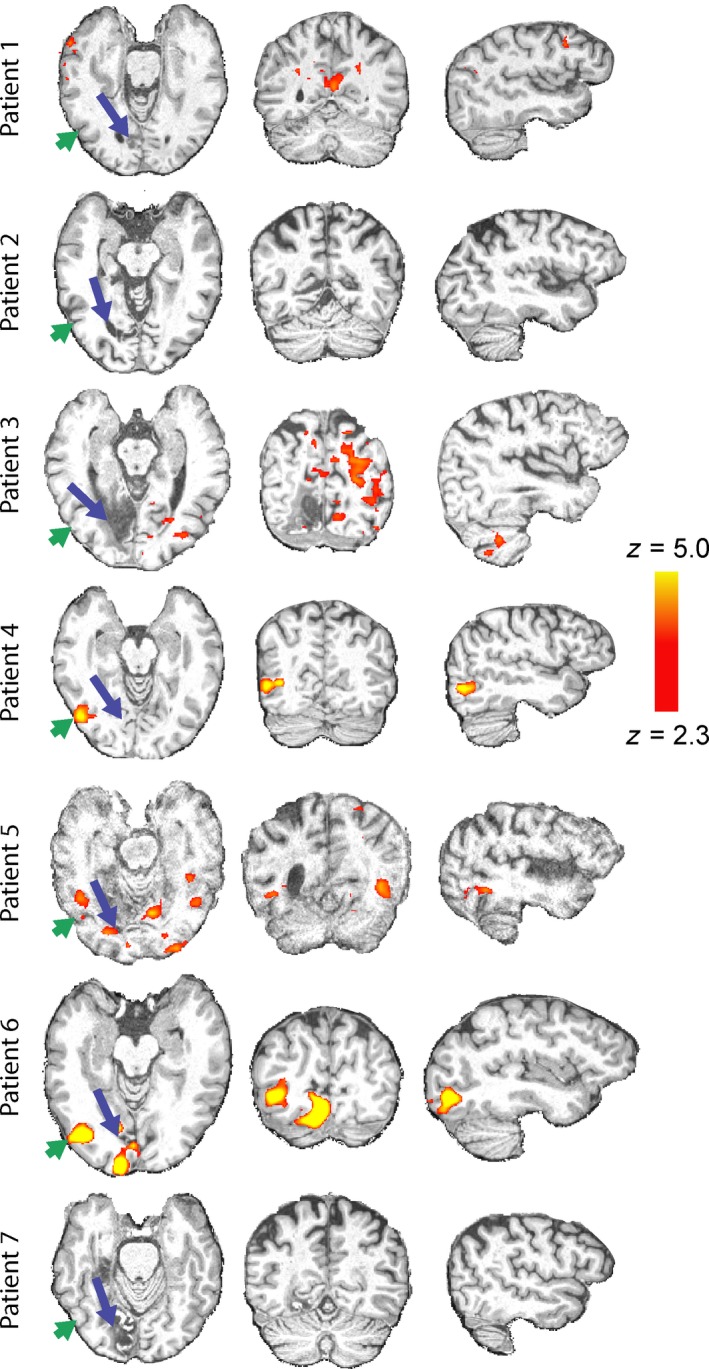
Activation to coherent dot movement compared to random noise at first scanning session. The stimulus was presented to the cortically blind hemifield. Only three patients (4, 5 and 6) showed significant activity to this stimulus in the lesioned hemisphere. The blue arrows indicate the location of the lesion, in all cases shown on the left side for illustration purposes. The green arrow indicates the location of putative hMT+. See *Table* 1 for actual lesion hemisphere.

To determine whether visual motion information can be relayed to the extrastriate cortex when V1 is damaged, the fMRI response to coherence motion was contrasted with the response to the noise stimulus. *Figure* 3 shows the brains of each of the patients with the blue arrow indicating the lesion location. In each case the lesion is shown on the left of the figure, although this is for illustration purposes only. The hemisphere that is actually damaged is given in *Table* 1 and the visual field deficit can be seen in *Figure* 1. Visual stimulation was in the impaired hemifield. Only three of the patients show activity in hMT+, which suggests that the likelihood of residual vision may be relatively low. Patient 5 showed excessive head motion (up to 4 mm) in each scan run, so was not included in any of the group analyses because much of the BOLD activity was likely to be artefactual.

**Table 1:** Patient clinical characteristics

	Gender	Age at study	Side	Duration (months)	Lesion size (mm^3^)	tDCS
Patient 1	M	46	Left	6	7720	Anodal
Patient 2	M	61	Right	15	7230	Sham
Patient 3	F	71	Right	21	25 710	None
Patient 4	F	49	Right	26	1320	Sham
Patient 5	M	76	Left	36	21 020	Sham
Patient 6	F	26	Left	18	3290	Anodal
Patient 7	M	62	Right	15	25 170	Anodal

### Across the entire group activation to coherently moving dots was reduced outside of the visual cortex between the two scan sessions

**Figure 4:**
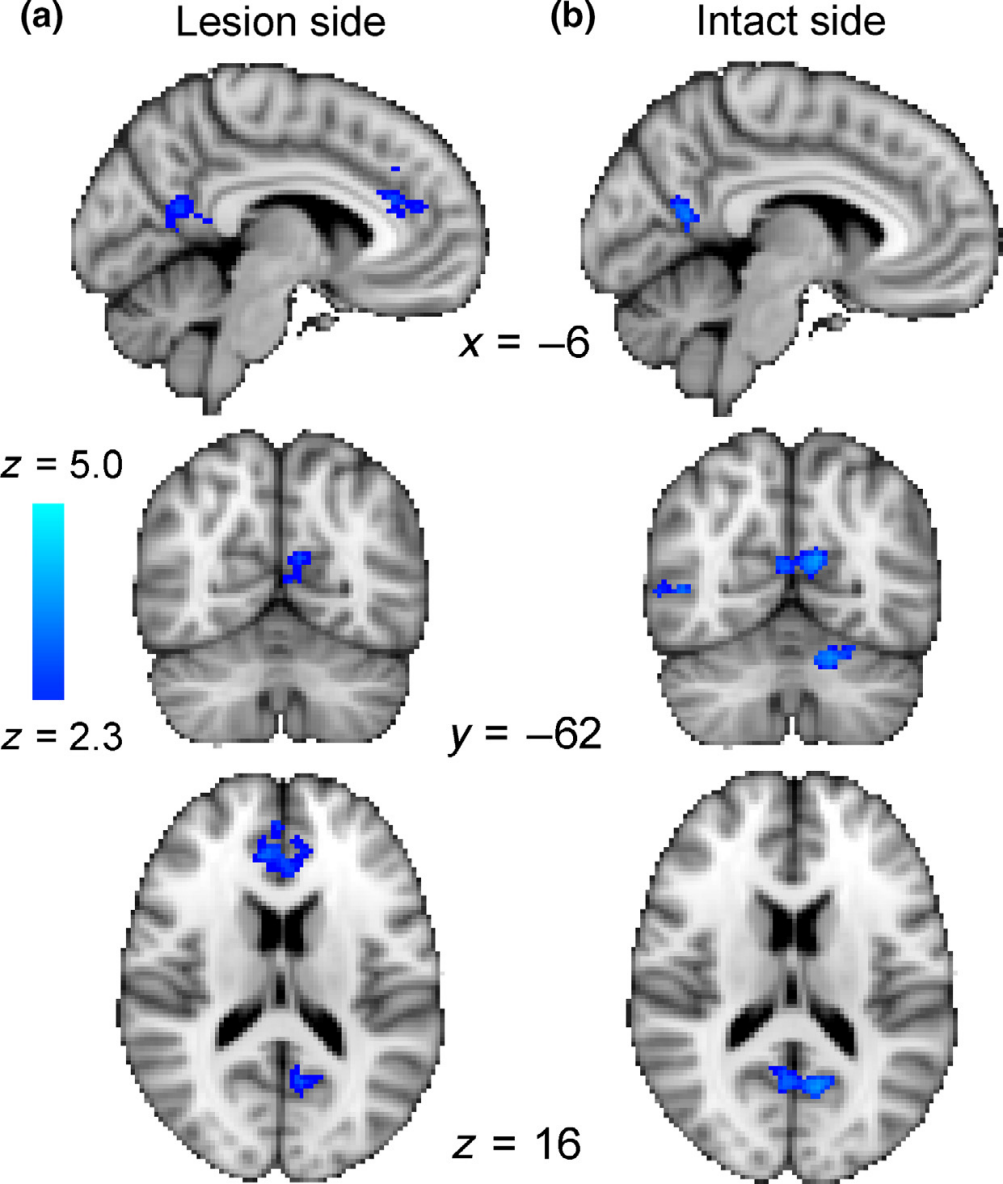
A comparison of the pre‐ and post‐training scan sessions across all patients indicates that there was reduced activity in the inferior part of the precuneus in the second scan whether the stimuli were presented to the impaired (a) or intact (b) hemifield.

While patients were trained on the visual motion discrimination task only in their blind hemifield, during the fMRI session visual stimuli were presented individually to both the blind and intact fields. *Figure* 4 shows the change in activity across the whole group after the 5 days of training when stimuli are presented to the impaired (a) and intact (b) hemifields. Activation was reduced in the inferior part of the precuneus irrespective of whether the stimuli were presented to the lesioned or intact fields.

### Following training, ipsilateral activity in hMT+ was reduced

**Figure 5:**
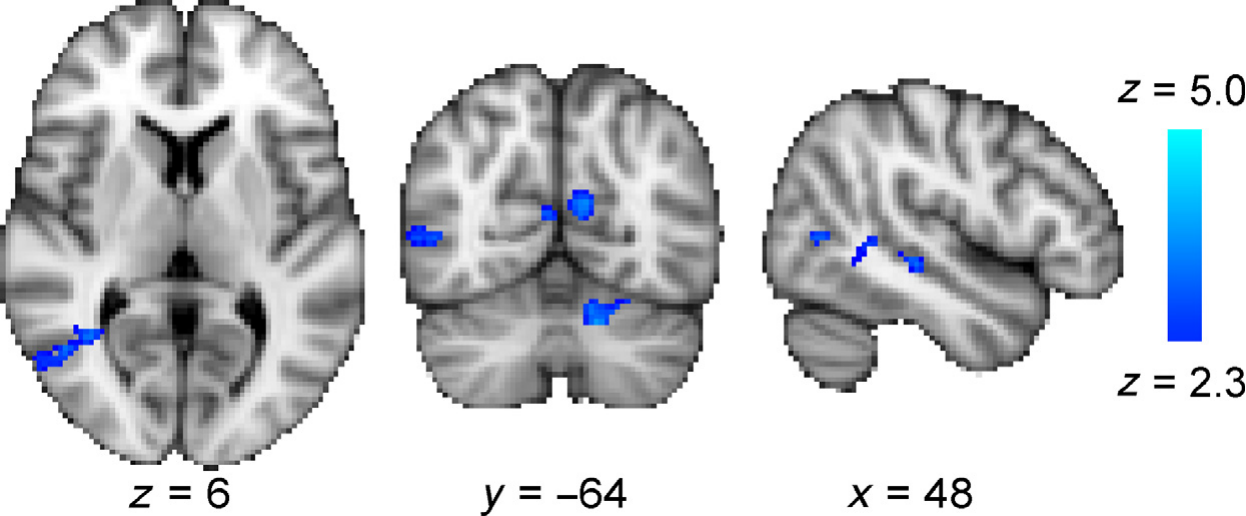
Across the patient group, training led to a reduction in activity in hMT+ on the lesioned side of the brain when the stimulus was presented to the sighted hemifield. This indicates a reduction of ipsilateral activity in the lesioned hemisphere following training. Similarly, there is a region of the cerebellum that also shows a reduction in activity following training. As would be expected, this change is contralateral to the cortical change in hMT+.

In contrast to the temporo‐parieto‐occipital junction there was a change in the visual cortex only when stimuli were presented to the intact hemifield. *Figure* 5 shows the decrease in activity in hMT+ in the lesioned side of the brain. The receptive fields in hMT+ are relatively large and, particularly in MST, can include ipsilateral representations. Thus, it is not surprising that there is activity in the lesioned hemisphere, although it is not clear why the activity should decrease after training.

**Figure 6:**
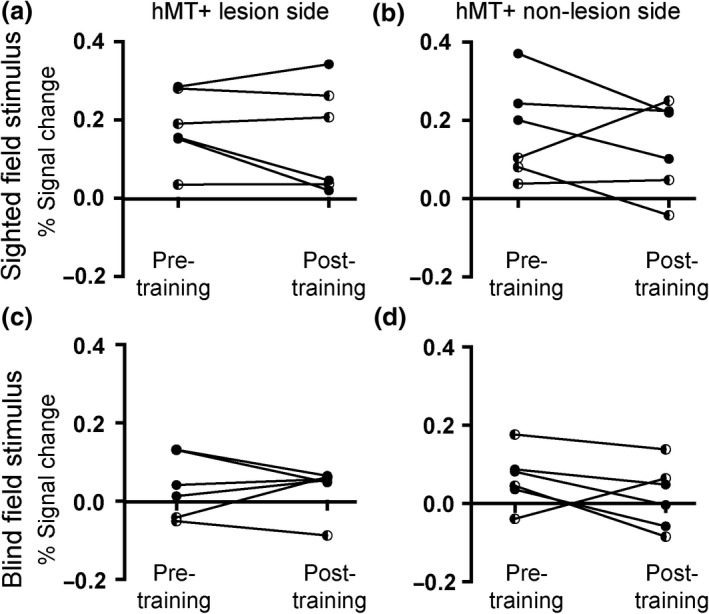
The signal change in hMT+ does not differ significantly between the two scan sessions whether stimuli were presented to the trained (blind) hemifield or the untrained (sighted) hemifield. In each case, the data from Patient 5 have been removed as they did not reflect reliable activity due to the excessive motion during the fMRI scans. Patients who received anodal tDCS are shown with the half‐filled symbols.

A region of interest analysis to quantify the signal change in area hMT+ showed that of the six patients with reliable BOLD activity, four showed a decrease in activity in the post‐training scan in response to stimulation of the intact hemifield (*Figure* 6 *a*). There was no significant change in BOLD signal across the group. Furthermore, there was no correspondence between the patients who received tDCS and change in response. *Figure* 6 *b* shows the response in hMT+ of the intact hemisphere, where minimal changes following training were identified. When the stimulus was presented to the impaired hemifield the responses in hMT+ on both sides were reduced compared to the intact hemifield (*Figure* 6 *c,d*), and there were no significant differences between BOLD signal pre‐training compared to post‐training.

To visualise these changes in BOLD response as brain activation, the patterns to coherent motion are shown pre‐ and post‐ training for each of the six patients individually in *Figures* S1 and S2.

### Patients receiving anodal tDCS showed a different response to those who did not

**Figure 7:**
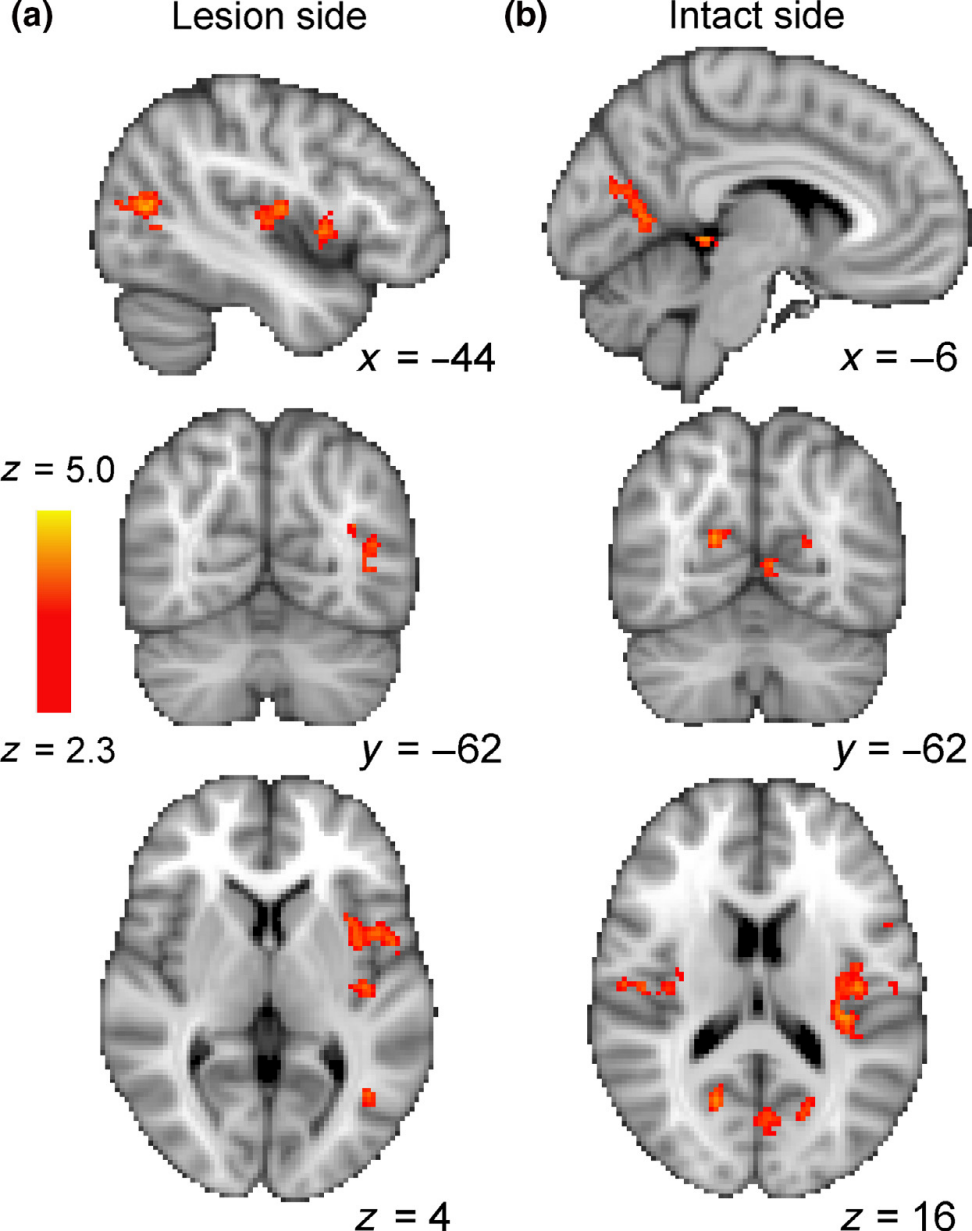
A preliminary analysis of the three patients receiving anodal vs the three patients with sham or no tDCS indicated that there was a difference in the BOLD change before and after training. With the stimulus in the blind field, projecting to the lesion side (a), the group that received anodal tDCS showed less reduction in BOLD signal in healthy hMT+ and in the insula. When the stimulus was presented to the healthy visual field (b), there was less reduction in the occipito‐parietal junction and the insula.

The comparison of patients receiving anodal tDCS compared to sham or no tDCS needs to be interpreted with considerable caution, since there were only three patients in each group. *Figure* 5 shows the regions in which the BOLD signal was reduced after training. *Figure* 7 shows the brain regions in which the group receiving tDCS stimulation showed less reduction in BOLD after training than those who received sham or no stimulation. When stimuli were presented to the visual field projecting to the lesioned side of cortex (A), area hMT+ in the healthy side showed a difference between the two groups. When stimuli were shown to the sighted field (B), there was a difference around the occipito‐parietal junction, and in both cases, there was a difference in the insula. Even though these analyses were performed using a mixed‐effects design that takes into account inter‐subject variability, the small numbers mean that an individual patient could have a significant effect on the results.

## Discussion

The main finding from this pilot study is that, unlike healthy controls,^17^ training of a motion direction discrimination protocol for 5 days did not improve performance in patients who are cortically blind. In this study, we did not train the sighted hemifield, so we cannot explicitly exclude the possibility that the lack of learning is a general phenomenon. However, this seems unlikely, since in our previous studies all participants showed improved performance following a similar training regime.^17^, ^18^

### Healthy control participants do not show a decrease in BOLD signal after training

There was a decrease in BOLD activity in hMT+ in the lesioned hemisphere when motion stimuli were presented to the intact visual hemifield. For this study, there was no control group of patients with hemianopia who did not receive visual training. Thus, it is not possible to state conclusively that the change in signal was due to the training. However, our previous study using the same training protocol (without tDCS) in healthy participants did not show this decrease in BOLD activity in either the trained group or a control group who were scanned twice but received no training.^18^ Rather, they showed an increase in activity in neighbouring area MST in the trained hemisphere which correlated with the amount of learning.

One explanation for this finding might be that the visual motion areas adapt to the visual training in different ways, depending on the inputs. In the motor system, there is considerable evidence that stroke to motor areas is associated with increased activation in motor areas ipsilateral to the affected limb e.g.,^29^ although it is still not clear whether this is adaptive or maladaptive. The data presented here are consistent with previous data suggesting that there is considerable ipsilateral activation in hMT+ of the lesioned hemisphere when moving stimuli are presented to the sighted hemifield.^30^ In the future, it will be necessary to determine whether this short‐term reduction in ipsilesional activity in the damaged hemisphere after training has any relationship to long term improvement in visual function.

### Long‐term training

The lack of change of visual motion discrimination in any of the patients provides clear evidence that this short duration of training on a motion direction discrimination protocol is not sufficient to improve visual perception in the blind hemifield of patients with hemianopia, even with the addition of tDCS. This contrasts starkly with data from healthy participants who show improvement in visual performance following the same protocol without tDCS.^18^ Since longer term training has been demonstrated to improve performance,^13^, ^14^, ^15^ it is likely that the response to training is much slower in patients. It is possible that the neural changes found in hMT+ following the short‐term training may be beneficial for much longer term improvement in visual perception. As mentioned above, testing this hypothesis will require imaging data acquired longitudinally over longer‐term training.

### The cerebellum shows a change in activity following training

In addition to the reduction in activity in hMT+ in the lesioned side of the brain, there was a region of reduced activity in the cerebellum. The change in the cerebellum was located in the cerebellar hemisphere contralateral to the cortical hMT+ change, as would be predicted given the ipsilateral organisation of the cerebellum. Furthermore, the region showing this change was centred on lobule VI, a region shown to be involved in processing of visual motion stimuli in healthy participants.^31^ Activation of this region in healthy participants is supported by studies of patients with cerebellar lesions, who are impaired in making judgements about visual motion direction, in addition to showing abnormal cortical visual responses measured with magnetoencephalography.^32^ Moreover, patients with cerebellar damage located in the posterior lobe show deficits in visual perceptual learning.^33^ Thus, the finding that the cerebellum shows a similar type of change in activity as area hMT+ is consistent with findings both in the healthy brain and the effects of cerebellar damage.

### Transcranial direct current stimulation does not affect visual performance, but does appear to affect BOLD signal changes

Non‐invasive direct current stimulation has been shown to lead to additional improvement in motor performance both in healthy participants and when used as an adjunct to physiotherapy in stroke rehabilitation.^34^ However, the effects of tDCS on visual performance appear to be considerably less clear. Early studies showed that cathodal stimulation of hMT+ improved visuomotor co‐ordination,^21^ while either anodal or cathodal reduced the motion after‐effect.^35^ An improvement in acuity has recently been demonstrated following anodal tDCS,^36^ but another study did not find any effect of tDCS on visual cortex excitability.^37^

While there have been single case studies of tDCS stimulation in patients with hemianopia,^38^ and a proposed clinical trial,^39^ there is currently little data to determine how beneficial tDCS might be for visual rehabilitation. The current study is preliminary, but none of the three patients who received anodal tDCS showed any improvement in visual motion direction discrimination. Compared to standard visual learning protocols, the current one was very short, only 5 days, and it may be that extended periods of tDCS may boost any visual learning. This remains to be determined in longitudinal studies.

The very preliminary analysis comparing the fMRI data between the anodal tDCS and sham/notDCS groups indicated that reductions in BOLD signal induced by training in several regions were lessened by anodal tDCS. One interesting feature is that the difference in hMT+ is predominantly in the healthy side of the visual cortex. This could be interpreted as an increase in the ipsilateral response following tDCS which, in the longer term, could boost visual performance. However, at this time, such a conclusion is speculative. The finding of differences between the two groups using a relatively conservative mixed‐effect analysis suggests further exploration of this approach in a larger study would be worthwhile, and would improve the interpretability of our results.

One issue to consider for future studies is whether sham stimulation provides a sufficient control for anodal tDCS. In some studies sensory side effects of real tDCS can be more frequent and severe than sham.^40^

### Activity is decreased in decision‐making areas following training after visual stimulation of either hemifield

While the visual rehabilitation training is designed to engage visual areas, any task that requires a response will clearly activate a network of areas including decision‐making areas and motor output. Indeed, non‐human primate studies of visual perceptual learning have shown that there are changes in the responses of lateral intraparietal area (LIP) neurons rather than those in visual cortex.^41^, ^42^ A previous study using the same learning protocol applied to healthy participants indicated increases in BOLD activity in the hippocampus, dorsolateral prefrontal cortex and frontal pole.^18^ In the current study, the patients showed a *decrease* in activity between the two scans in the occipito‐temporal junction in the lesioned hemisphere. This is a result that has been previously demonstrated in other studies of visual perceptual learning,^43^ and is suggested to reflect the reduction in attention demands following training. It is interesting that this should also be the case in the current patients, however, even when task performance does not improve.

### Eye movements do not improve performance

An eye tracker was used for most of the patients, but obtaining consistent, high quality recordings was challenging both inside and outside of the scanner. The experimenter watched the patients throughout the behavioural testing and the eye trace during the scanning to ensure they were following the instruction to fixate centrally. Most of the unusable data was due to the trace being lost, due to insufficient line of sight to the eye or excessive blinking. Participants did not improve on the motion task delivered here, regardless of eye movements that may have occurred and gone undetected due to unusable data.

## Conclusion

This pilot study indicates that, even though there was no improvement in visual performance, there are changes in the neural responses of visual areas, specifically hMT+, following motion direction discrimination training, and an indication that anodal tDCS can interact with these changes. It is possible that the neural changes could facilitate visual rehabilitation that is seen in longer term training protocols, perhaps reflecting the early stages of such training. Imaging at regular periods during rehabilitation is required to determine how any neural changes adapt over time as visual function improves.

## Disclosure

The authors report no conflicts of interest and have no proprietary interest in any of the materials mentioned in this article.

## Supporting information


**Figure S1.** In each patient the upper row shows the activation (red‐yellow) to visual stimulation in the sighted hemifield prior to training.
**Figure S2.** As in *Figure* 6, the upper row shows the activation (red‐yellow) to visual stimulation in the sighted hemifield prior to training.
