# Supplementary material for: Visual training in hemianopia alters neural activity in the absence of behavioural improvement: a pilot study
Source: Ophthalmic Physiol Opt. 2018 Oct 24;38(5):538–49. doi: 10.1111/opo.12584 (PMC6282990; doi:10.1111/opo.12584)
Supplement: Supplementary file 1 — Figure S1. In each patient the upper row shows the activation (red-yellow) to visual stimulation in the sighted hemifield prior to training. Figure S2. As in Figure 6, the upper row shows the activation (red-yellow) to visual stimulation in the sighted hemifield prior to training. [file 44402_2018_3805008_MOESM1_ESM.docx]

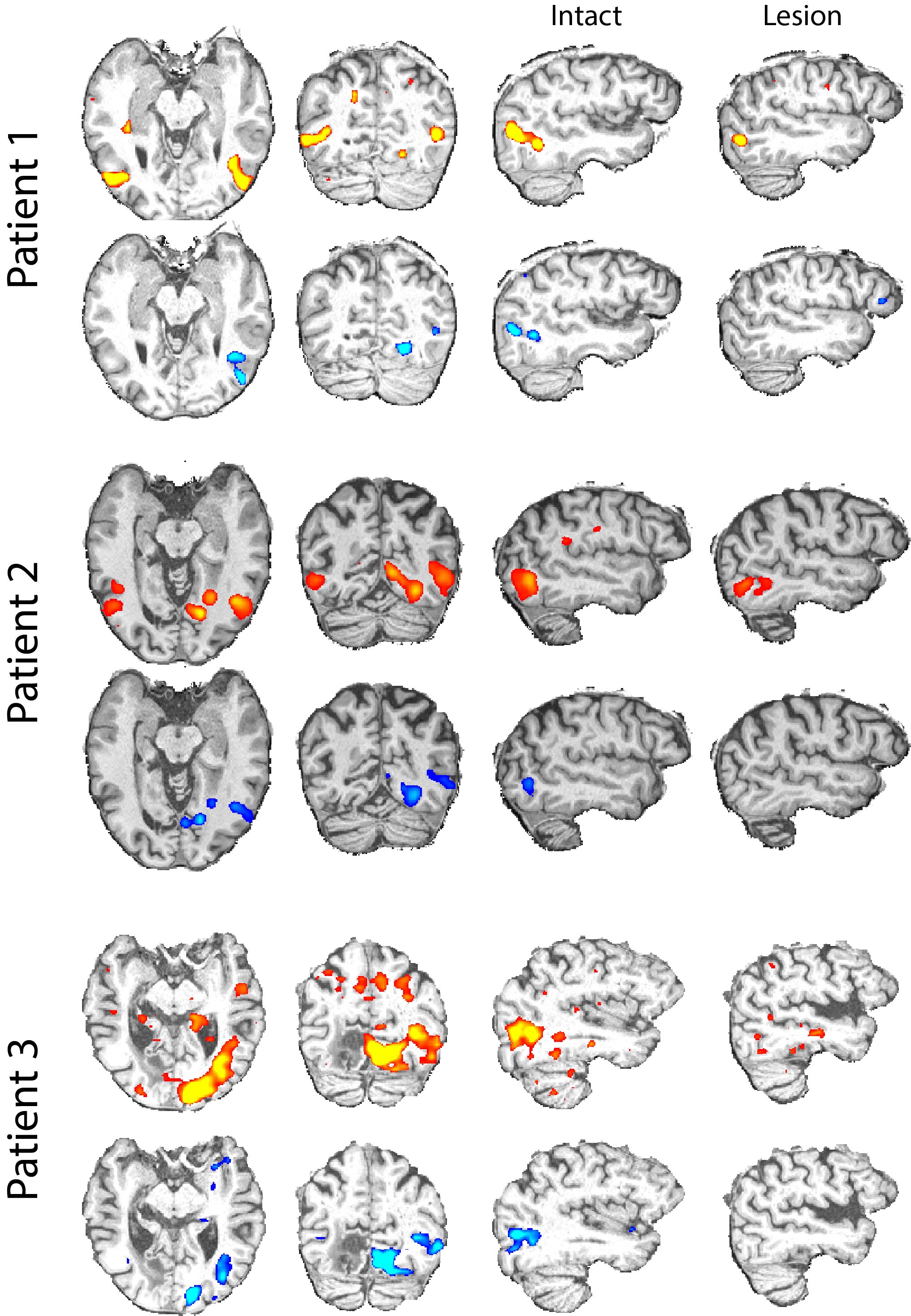


Supplementary Figure 1. In each patient the upper row shows the activation (red-yellow) to visual stimulation in the sighted hemifield prior to training. The lower row shows the same activation (blue) in the scan following training. Note that the ipsilateral activity in the lesioned hemisphere is no longer present post-training.


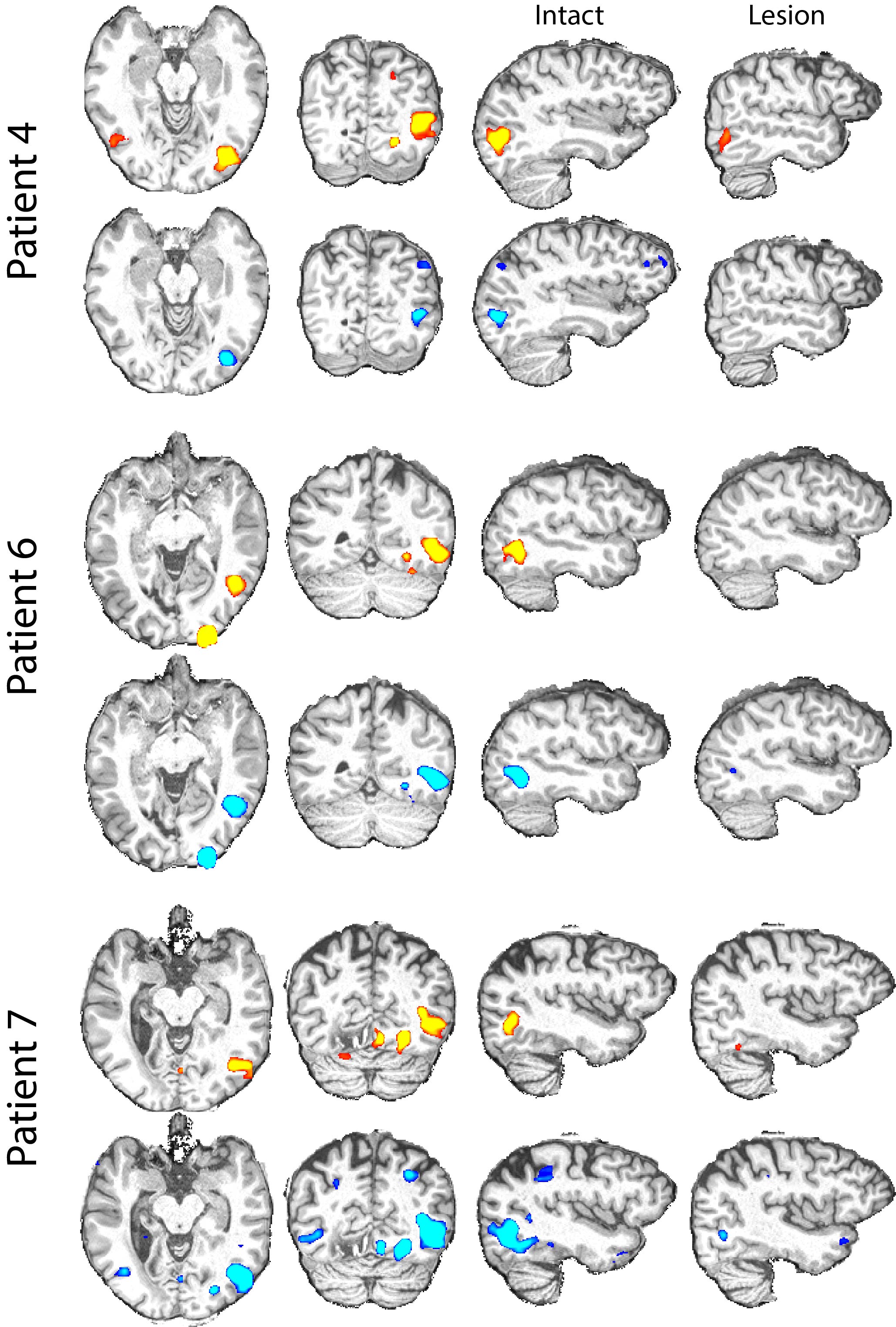


Supplementary Figure 2. As in Figure 6, the upper row shows the activation (red-yellow) to visual stimulation in the sighted hemifield prior to training. The lower row shows the same activation (blue) in the scan following training. Note that the ipsilateral activity in the lesioned hemisphere of Patient 4 is no longer present post-training. Patient 6 does not show any ipsilateral activity in either scan session and Patient 7 shows the opposite pattern to the other patients, with an increase in ipsilateral activity after training.
